# Predictive Ability of Self Compassion in Psychosocial Outcomes of Caregivers of Persons With Dementia

**DOI:** 10.1093/geroni/igab046.3519

**Published:** 2021-12-17

**Authors:** Claire Grant, Katherine Judge

**Affiliations:** 1 Cleveland State University, South Euclid, Ohio, United States; 2 Cleveland State University, Cleveland, Ohio, United States

## Abstract

Caregivers of Individuals with Dementia (IWDs) often face increased rates of depression, anxiety, and burden because of their role as caregiver. Self-compassion, a construct centered around self-kindness and understanding has not been well studied within the caregiving population. The present study was aimed at understanding the relationship between self-compassion and the psychosocial outcomes of burden, depression, and anxiety. Strong relationships between self-compassion and these outcomes have been established in other populations, but these relationships have not been studied with the dementia caregiving population. A diverse sample of dementia caregivers providing over 5 hours of care per week were recruited through CloudResearch and MTurk (N = 99). Participants were aged 18 to 69 years (M = 38.61) and 66.7% were female. 67.7% were White, 13.1% were Black, and 8.1% were Asian. 73% were children/in law or grandchildren/in law of the individual with dementia and 12% were a close friend of the individuals with dementia. The individuals with dementia had an average age of 73.88 years. Results of multiple regression models showed that self-compassion was a significant predictor of depression (β = -.25, p = .025), anxiety (β = -.36, p = .001), and burden (β = -.25, p = .023) even while controlling for other constructs including self-esteem, types of coping, and IWD impairment level. Self-compassion will be discussed as a novel contribution to the caregiving literature in furthering our understanding of well-being predictors and how to target self-compassion as a modifiable factor for offsetting the negative impacts of caregiving.

